# Nonlinear optimization for a low-emittance storage ring

**DOI:** 10.1107/S1600577524004569

**Published:** 2024-06-25

**Authors:** Bonghoon Oh, Jinjoo Ko, Seunghwan Shin, Jaehyun Kim, Jaeyu Lee, Gyeongsu Jang

**Affiliations:** a Department of Accelerator Science Korea University 2511 Sejong-ro Sejong30019 South Korea; b Pohang Accelerator Laboratory, POSTECH, Pohang, Kyungbuk37673, South Korea; c Mutipurpose Synchrotron Radiation Construction Project, Korea Basic Science Institute, 162 Yeongudanji-ro, Cheongwon-gu, Cheongju, Chungcheongbukdo28119, South Korea; SESAME, Jordan

**Keywords:** storage ring, dynamic aperture, MOGA, MGGOP, self-adaptive crossover

## Abstract

A multi-objective genetic algorithm (MOGA) is a powerful global optimization tool, but its results are considerably affected by the crossover parameter η_c_. Finding an appropriate η_c_ demands too much computing time because MOGA needs be run several times in order to find a good η_c_. In this paper, a self-adaptive crossover parameter is introduced in a strategy to adopt a new η_c_ for every generation while running MOGA. This new scheme has also been adopted for a multi-generation Gaussian process optimization (MGGPO) when producing trial solutions. Compared with the existing MGGPO and MOGA, the MGGPO and MOGA with the new strategy show better performance in nonlinear optimization for the design of low-emittance storage rings.

## Abstract 2

A self-adaptive crossover parameter is introduced in a strategy to adopt a new η_c_ for every generation while running a multi-objective genetic algorithm.

## 1. Introduction

In the design of an accelerator complex, nonlinear beam dynamics optimization is often repeatedly conducted to ensure the accelerator meets the required performance. A larger dynamic aperture (DA) secures a high injection efficiency as it helps to capture the injected beam of large amplitude. A longer Touschek lifetime implies that the loss of stored beam due to energy transfer between electrons in the beam is minimized. The DA area and Touschek lifetime can be increased by imposing appropriate sextupole strengths. Optimization algorithms can help to find suitable sextupole strengths.

Several stochastic global optimization schemes such as the multi-objective genetic algorithm (MOGA) (Deb *et al.*, 2002 ▸; Yang *et al.*, 2009 ▸), multi-objective particle swarm optimization (MOPSO) (Pang & Rybarcyk, 2014 ▸; Lin *et al.*, 2015 ▸) and multi-generation Gaussian process optimization (MGGPO) (Song *et al.*, 2020 ▸) have been successfully adopted in optimizing the lattice design.

MOGA exploits survival of the fittest by analogy with biological evolution. First, *N* candidate solutions are produced randomly. These parent solutions are combined to yield child solutions by crossover, and some variables of child solutions are changed by mutation. The *N* best, *i.e.* fittest, solutions are retained and others are abandoned so that a population of solutions is maintained. These solutions become parents of the next generation. This process is carried out iteratively until the *N* solutions converge sufficiently (Pareto front).

In contrast, MGGPO generates more solutions (trial solutions) than the population by utilizing crossover and mutation of MOGA and random movement using the MOPSO mechanism which mimics animals’ organized movement such as flocking behavior to find food. MGGPO uses Gaussian process (GP) regression, a kind of supervised machine learning, to predict objectives of trial solutions because this prediction requires negligible computing time. The *N* best solutions are retained and others are discarded. Objectives of selected solutions are evaluated to establish a GP model for GP regression in the subsequent generation.

In MOGAs, crossover, *i.e.* simulated binary crossover (SBX), and mutation are quantified using the crossover parameter η_c_ and the mutation parameter η_m_; these parameters affect the Pareto front and the convergence speed. In existing MOGAs, η_c_ is fixed at every generation. In this paper, we introduce the self-adaptive crossover parameter (SAXP) scheme, which uses a new η_c_ at each generation (Deb *et al.*, 2007 ▸). A SAXP strategy was also adopted to a MOGA crossover for manufacturing trial solutions in MGGPO. Section 2 introduces the SAXP strategy. Section 3 compares the results of MGGPO and MOGA in optimizing nonlinear dynamics of low emittance storage rings, each using either a fixed crossover parameter (FXP) or SAXP scheme. Section 4 presents conclusions.

## 2. Self-adaptive crossover parameter strategy

We used a real-coded genetic algorithm (GA) for nonlinear beam dynamics optimization and, therefore, for crossover we used an SBX formula,
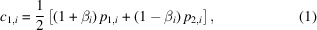

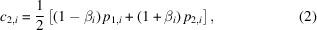
where *i* is the decision-variable index, *p*_1,*i*_ and *p*_2,*i*_ are decision variables of the parent solutions, *c*_1,*i*_ and *c*_2,*i*_ are decision variables of child solutions, and
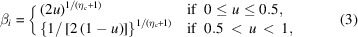
where 0 ≤ *u* ≤ 1 is a uniformly distributed random number. In MOGA, η_c_ is set by the user and generally remains constant during the whole MOGA process.

Equations (1) and (2) demonstrate that two child solutions *c*_1,*i*_, *c*_2,*i*_ lie on a line between two parent solutions *p*_1,*i*_, *p*_2,*i*_ in decision-variable space, and that the children are located inside a region bounded by parents if 0 ≤ β_*i*_ ≤ 1 (*i.e.* 0 ≤ *u* ≤ 0.5), and outside of that region if β_*i*_ > 1 (*i.e.* 0.5 < *u* < 1).

**Figure 1:**
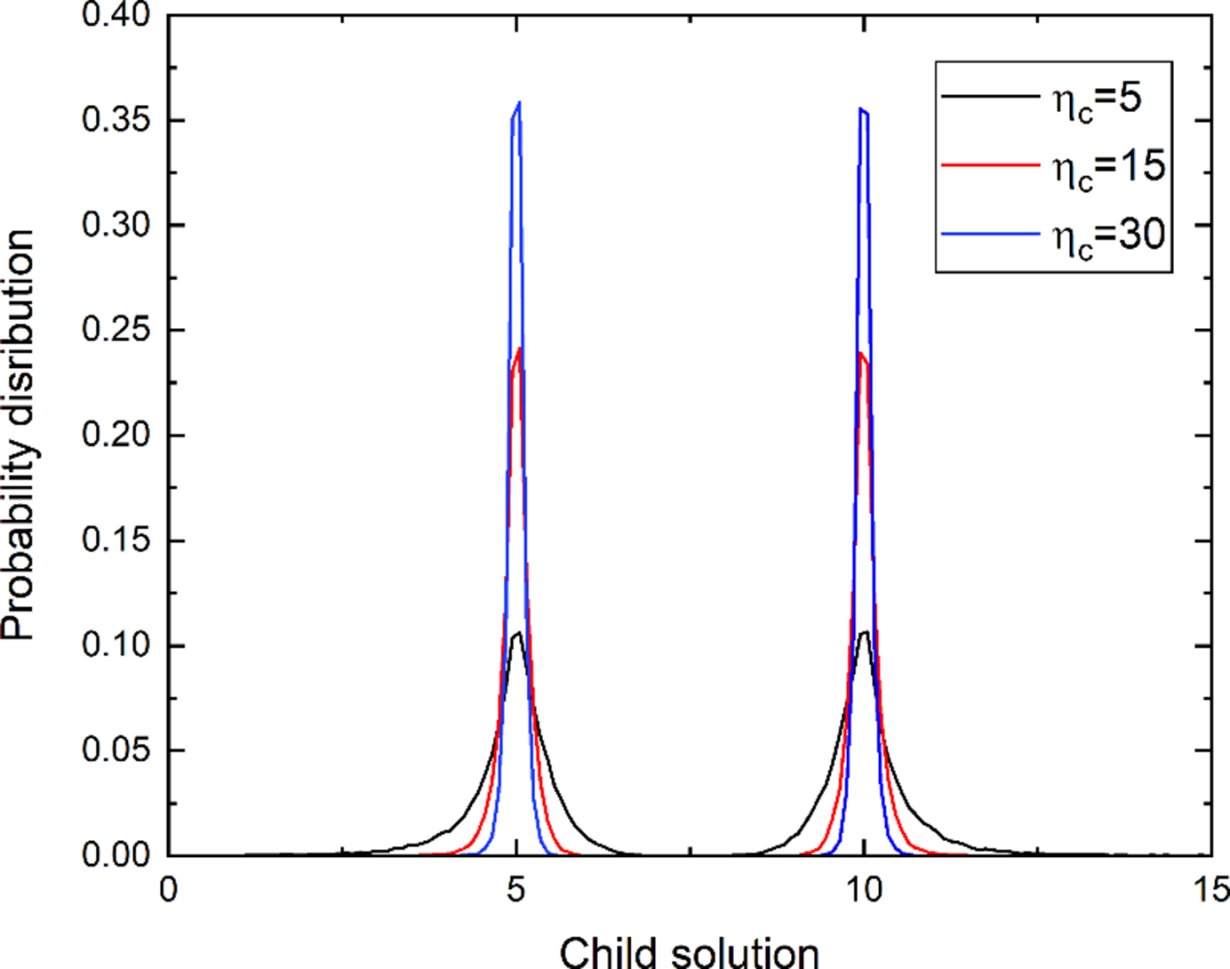
Distribution of two offspring according to the crossover parameter η_c_.

**Figure 2:**
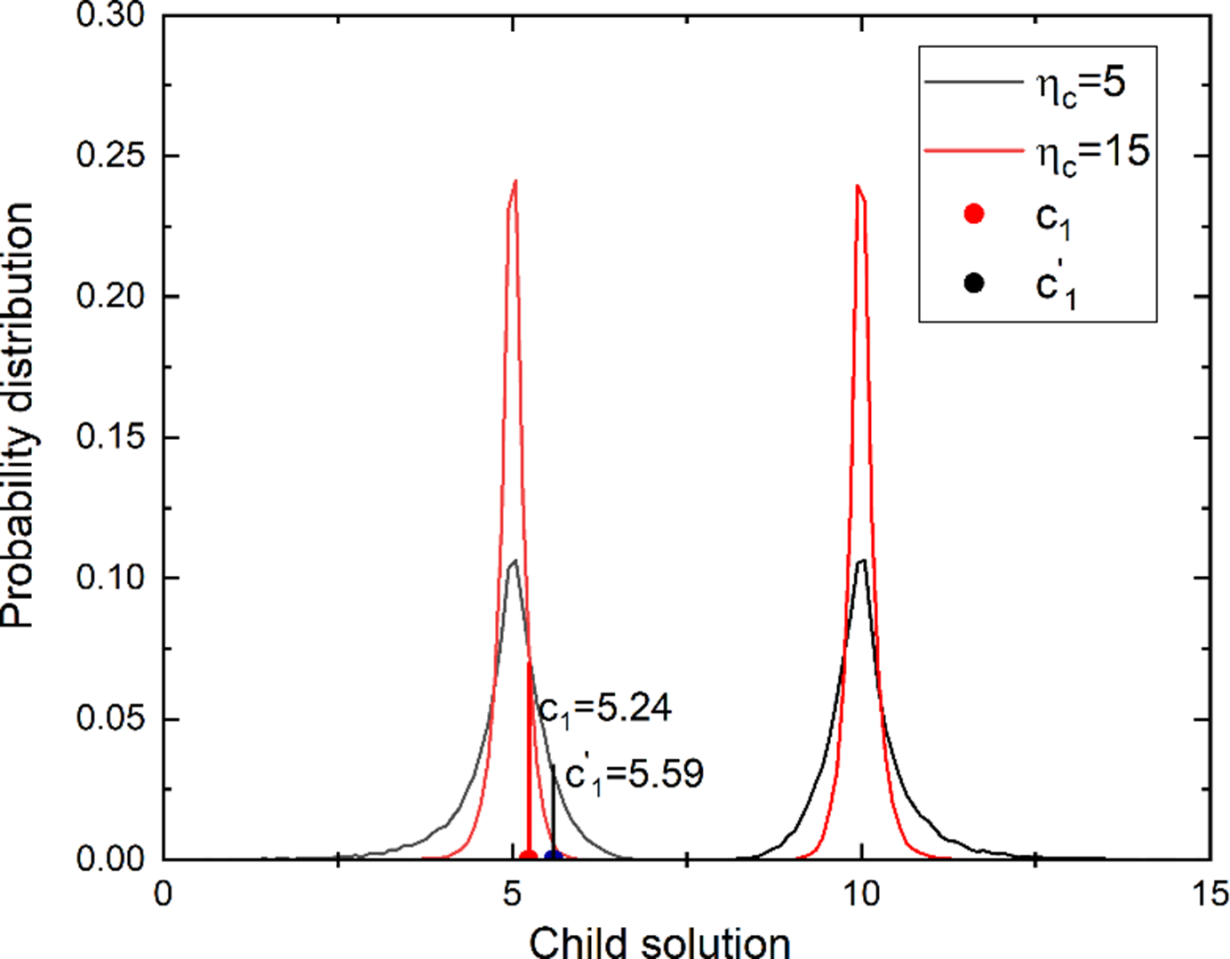
Locations of *c*_1_ and 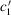 with η_c_ = 5 and 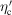 = 10 and the same random number *u* = 0.1.

η_c_ in equation (3) determines the probability distribution of offspring. As η_c_ increases, the probability distribution of offspring around the parents narrows in the variable space (Fig. 1 ▸); *i.e.* when random number *u* is fixed, the child solution nears the parent as η_c_ increases. For example, when *p*_1_ = 5, *p*_2_ = 10 and *u* = 0.1, the first child *c*_1_ created with η_c_ = 15 is 5.24 and the second child 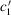 created with 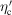 = 5 is 5.59 (Fig. 2 ▸). This characteristic can be exploited using the principle of Nelder and Meade’s simplex (Press *et al.*, 1992 ▸) to improve the child solutions. Firstly, offspring are produced by crossover using a fixed η_c_ that is pre-defined by the user. Objectives of each child and two parents are compared. If a child solution is better than both parent solutions, then a better solution than this child can probably be found at a farther position from the nearest parent than this child. This principle was created by exploiting the *expansion* concept of the simplex. To obtain this ‘farther’ solution, η_c_ is replaced with a new parameter 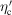 < η_c_ and another crossover is conducted. If the resulting child is inferior to the parents, then another crossover is conducted using 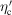 > η_c_ by exploiting *contraction* of the simplex. Otherwise, the crossover uses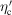 = 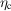. The same *u* must be used for the first and second crossovers.

The new 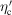 is chosen using a protocol. The relationship between η_c_ and β_*i*_ differs [equation (3)] according to whether 0 ≤ *u* ≤ 0.5 (0 ≤ β_*i*_ ≤ 1) or 0.5 < *u* < 1 (β_*i*_ > 1). The formula for 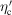 also differs according to whether the child solution is superior or inferior to the parent solutions. Therefore, four 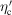 formulae will be listed.

When child *c*_1_ is superior to both parents *p*_1_ and *p*_2_, it must be that 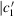 − *p*_1_| > |*c*_1_ − *p*_1_|. If β_*i*_ > 1, the child is located outside of the two parents by external division from equation (2). From equation (2), β_*i*_ can be written as
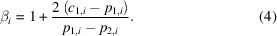
When β_*i*_ > 1, equation (3) can be rearranged to
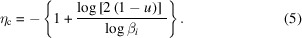
We introduce parameter
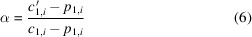
to quantify how much changing the crossover parameter affects the difference between *c*′ and *c*.

Here, 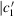 − *p*_1_| > |*c*_1_ − *p*_1_|, so α > 1. As α increases, 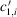 moves away from *p*_1_. From equation (4), 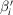 can be calculated by replacing *c*_1,*i*_ with 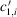,
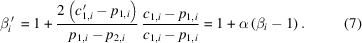
Using equation (5), the new 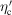 can be written as
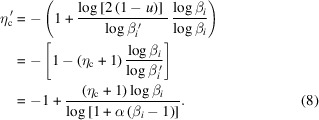
In equation (8), 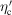 < η_c_ if α > 1 and β_*i*_ > 1. The right-hand side of equation (8) includes β_*i*_, which has decision variable index *i*; this condition means that 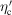 is determined differently according to all variables of all solutions. Strictly speaking, 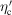 should be denoted as 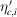. If child *c*_1_ is inferior to both parents *p*_1_ and *p*_2_, then α can be replaced with 1/α in equation (8) to make 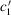 be created closer than *c*_1_ to *p*_1_. Therefore, 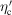 can be presented as
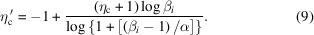
If 0 ≤ β_*i*_ < 1, offspring are between the two parents by internal division from equation (2). When β_*i*_ < 1, from equation (3),
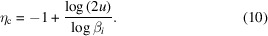
From equation (3), 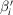 must not be negative. If we define 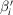 as equation (7), then 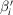 may be negative, when β_*i*_ < 1, because α can be any value > 1. We need a new α formula to assure that 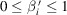 when 0 ≤ β_*i*_ ≤ 1. When *c*_1_ is superior to *p*_1_ and *p*_2_, increased α must move 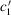 farther away from *p*_1_ than *c*_1_. Here, when 0 ≤ β_*i*_ ≤ 1 and *c*_1_ is superior to *p*_1_ and *p*_2_, then we can introduce α with the relationship
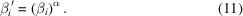
With equation (11), if 0 ≤ β_*i*_ ≤ 1, then 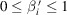. If α ≥ 1, then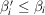 and, as α increases, 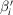 decreases. From equation (4), when 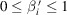, a decreased 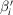 increases 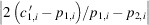 because 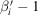 = 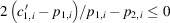; *i.e.* when α ≥ 1, 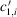 is produced at a farther position from *p*_1,*i*_ than *c*_1,*i*_, and when α increases, 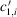 moves away from *p*_1, *i*_. Using equations (10) and (11),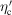 is calculated as
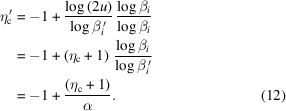
In equation (12), α > 1, so 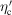 < η_c_. If *c*_1_ is inferior to *p*_1_ and *p*_2_, then we replace α with 1/α, so 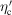 becomes
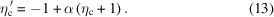
Otherwise, if a child solution is not inferior to and superior to both two parents, then we set 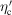 = 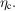 If 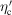 calculated using equations (8), (9), (12) and (13) is negative then we set 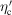 = 0. If α = 1, the scheme of SAXP is the same as that of FXP because 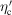 will always be the same as η_c_. Different 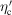 determined by equations (8), (9), (12) and (13) are adopted to each variable of each solution. This means that all variables of new child solutions are determined by the same crossover formulas equations (1)–(3) and different crossover parameter 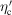.

## 3. Comparison of results of fixed and self-adaptive strategy

The SAXP scheme was first applied to MGGPO. MGGPO produces as many as 10*N* trial solutions by using random movement and 10*N* solutions by using crossover and mutation, where *N* is the population. Crossover can use the SAXP or the FXP scheme. To fulfill the SAXP strategy, objectives of child solutions must be calculated twice. This means that MGGPO using SAXP consumes double the computing time compared with MGGPO using FXP. However, first objectives investigation of the child solution to determine 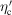 was not conducted using particle tracking but GP regression, meaning that not the calculation values but the expectation values of objectives were used to determine 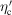. GP regression demands insignificant computing time, so MGGPO takes similar times when using SAXP and FXP.

**Figure 3:**
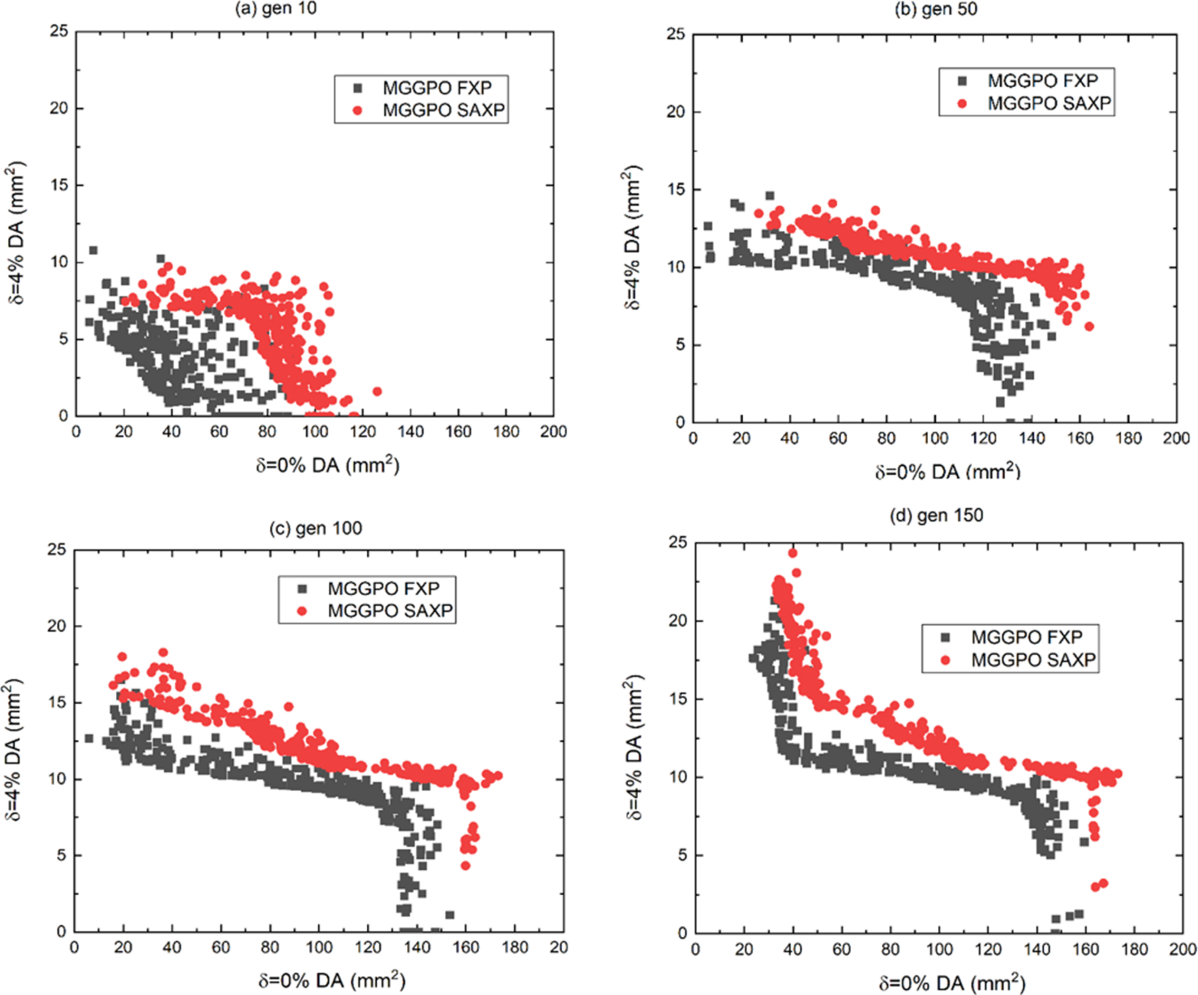
Comparison of objectives by MGGPO using FXP and SAXP with α = 3 at generation (*a*) 10, (*b*) 50, (*c*) 100 and (*d*) 150.

MGGPO exploiting both SAXP and FXP was used to simultaneously increase the on and off momentum dynamic apertures (DAs) of the Korea-4GSR lattice. Korea-4GSR is a 28-cell low-emittance electron storage ring with hybrid seven-bend achromat optics (Oh *et al.*, 2021 ▸). The ring has six sextupoles in each cell, and the ring is assumed to have a periodicity of 14 in terms of sextupole configuration (*i.e.* 12 power supplies for sextupoles in the ring). In the simulation, we used two of the 12 sextupoles to correct chromaticity and the remaining 10 sextupoles as decision variables of MGGPO. On (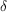 = 0%) momentum DA area and off (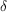 = 4%) momentum DA area were set as the two objectives. Population *N* was set at 300 and DA area was calculated using 100-turn tracking to reduce computing time. η_m_ = 60 and η_c_ = 60. For SAXP, α = 3 and 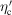 was calculated using equations (8), (9), (12) and (13). MGGPO with 100 generations was performed first using the MOGA crossover in the FXP way [Fig. 3 ▸(*a*)] then in the SAXP way [Fig. 3 ▸(*b*)], and the solutions were compared at the 100th generation in each case [Fig. 3 ▸(*c*)]. MGGPO with SAXP reveals better solutions than MGGPO with FXP [Fig. 3 ▸(*c*)].

MOGA using the SAXP and the FXP method was also performed, with the same decision variables and objectives that were used for MGGPO. Other values were *N* = 300, η_m_ = 25 and η_c_ = 10. The algorithm was run for 100 generations. When MOGA using SAXP is conducted, the objectives of both child solutions *c* and *c*′ were estimated using particle tracking, unlike in MGGPO, in which they were estimated by using expectation values of GP regression. For this reason, MOGA that used SAXP required twice as much computing time as MOGA that used FXP.

**Figure 4:**
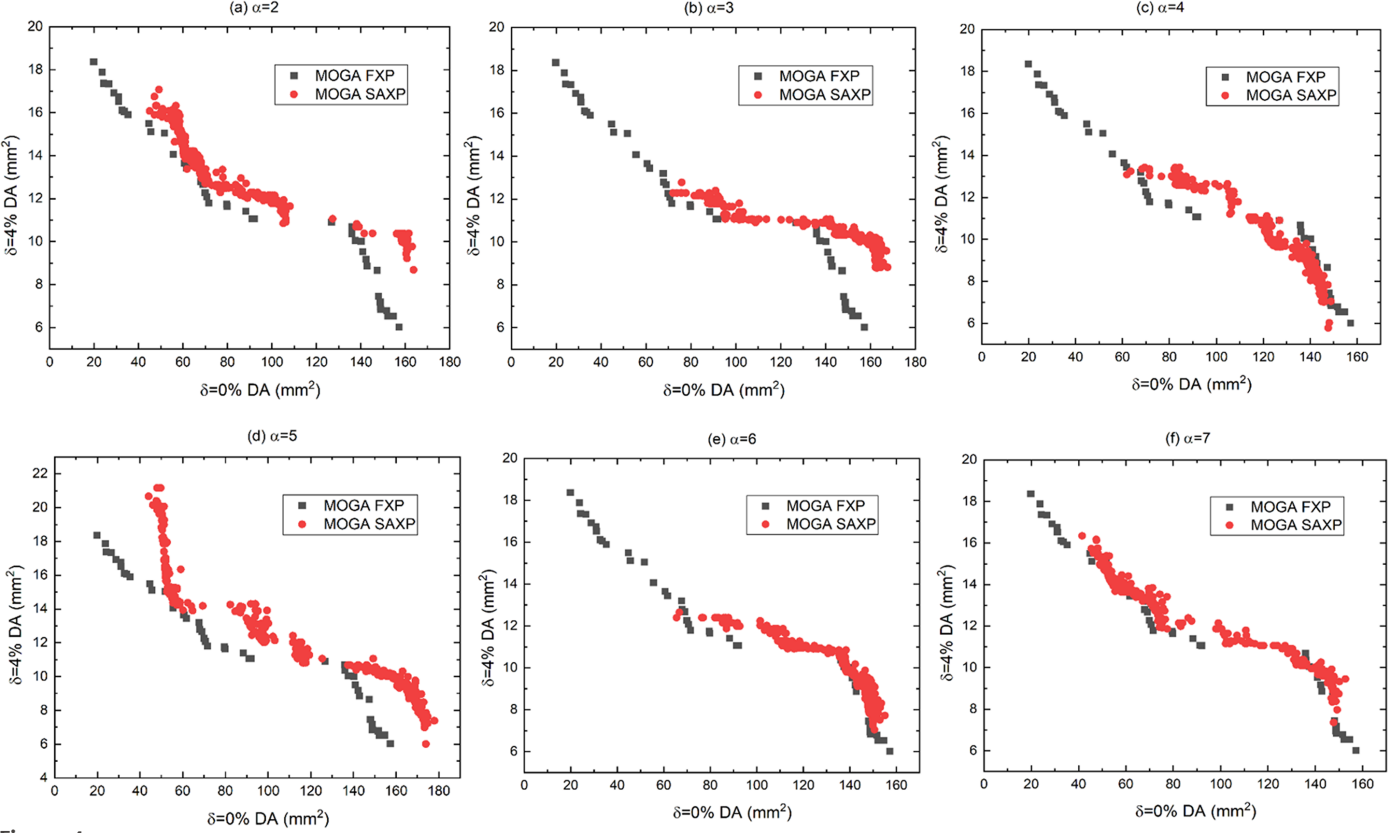
Comparison of objectives by MOGA using FXP and SAXP with (*a*) α = 2, (*b*) α = 3, (*c*) α = 4, (*d*) α = 5, (*e*) α = 6 and (*f*) α = 7.

**Figure 5:**
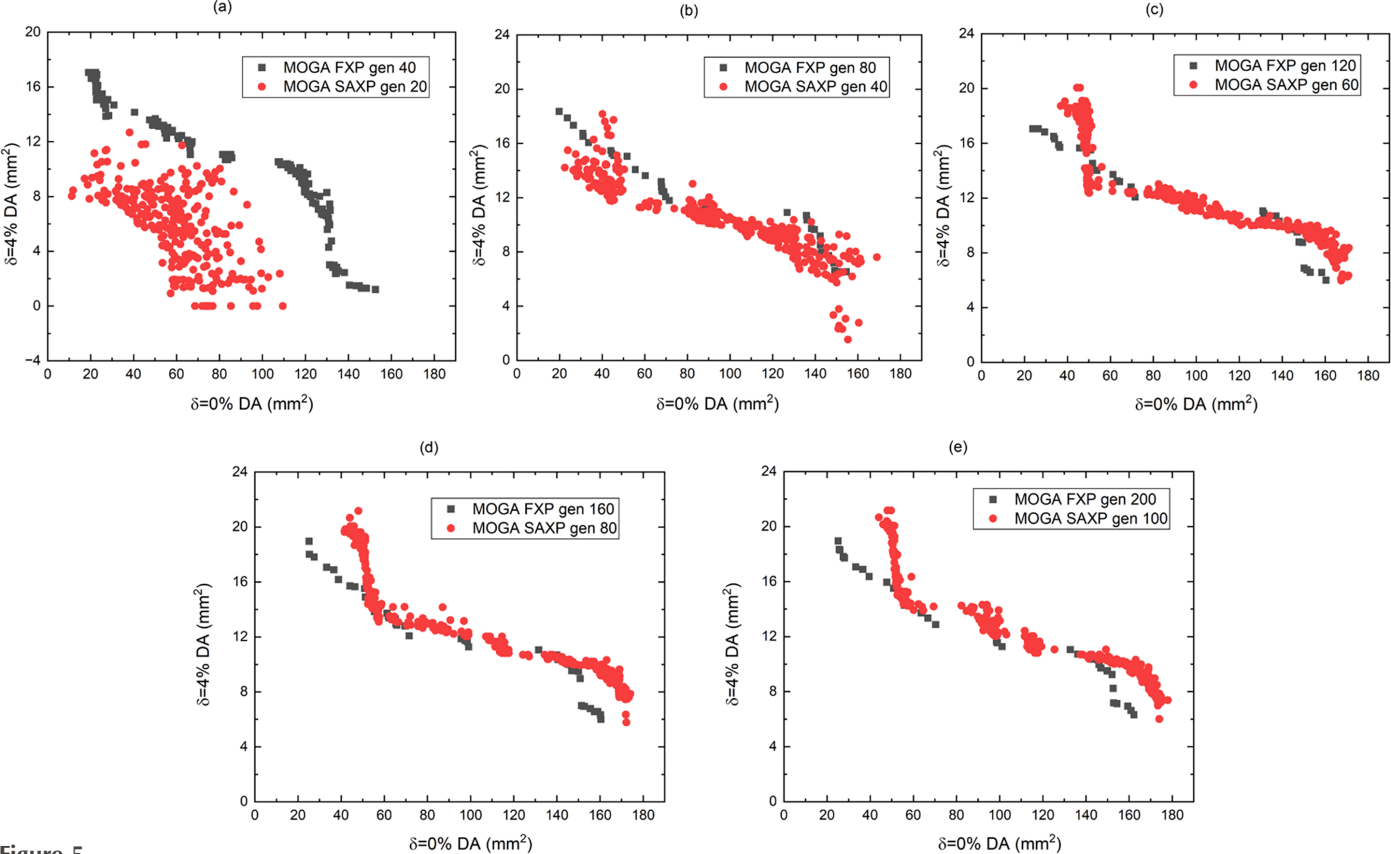
Results of MOGA with FXP strategy at 2*N* generations and SAXP strategy at *N* generations where *N* is (*a*) 20, (*b*) 40, (*c*) 60, (*d*) 80 and (*e*) 100.

**Figure 7:**
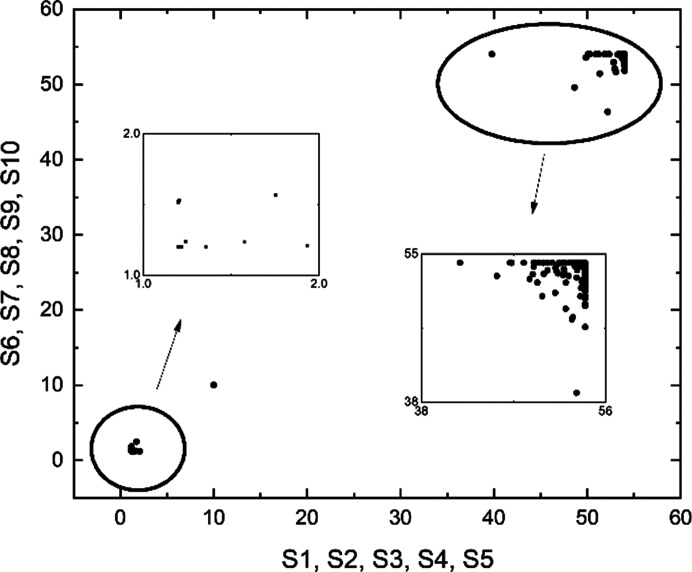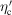 of all variables of all solutions obtained by the SAXP scheme with α = 5 at generation 100. S1–S10 are decision variables that represent 10 sextupole strengths.

First, to find an appropriate α value, MOGA with SAXP was conducted using a range of α [Figs. 4 ▸(*a*)–(*f*)]. Results indicated that α = 5 is a good choice for the SAXP scheme, and provides better solutions than MOGA using FXP. When α = 5, the results of MOGA using SAXP at *N* generations and FXP at 2*N* generations were compared with, *N* from 20 to 100, considering doubled computing time in the SAXP strategy compared with the FXP strategy (Figs. 5 ▸ and 6 ▸). Compared with the FXP scheme, the SAXP scheme gave inferior solutions at *N* = 20, 40 and similar solutions at *N* = 60, but superior solutions at *N* = 80, 100. The distribution of 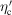 for all variables of all solutions obtained by the SAXP scheme with α = 5 at generation 100 is shown in Fig. 7 ▸.

## 4. Conclusion

Previous studies that have applied MOGA have usually used a constant crossover parameter for SBX, *i.e.* the FXP strategy. We tried another way for SBX by changing the crossover parameter at each generation, *i.e.* the SAXP strategy. We compared results of the MOGA optimization with the two schemes to simultaneously optimize the on- and off-momentum DA areas for the Korea-4GSR lattice. We verified that MOGA using SAXP certainly presents a better Pareto front than MOGA using FXP when an appropriate α value is adopted

## Figures and Tables

**Figure 6:**
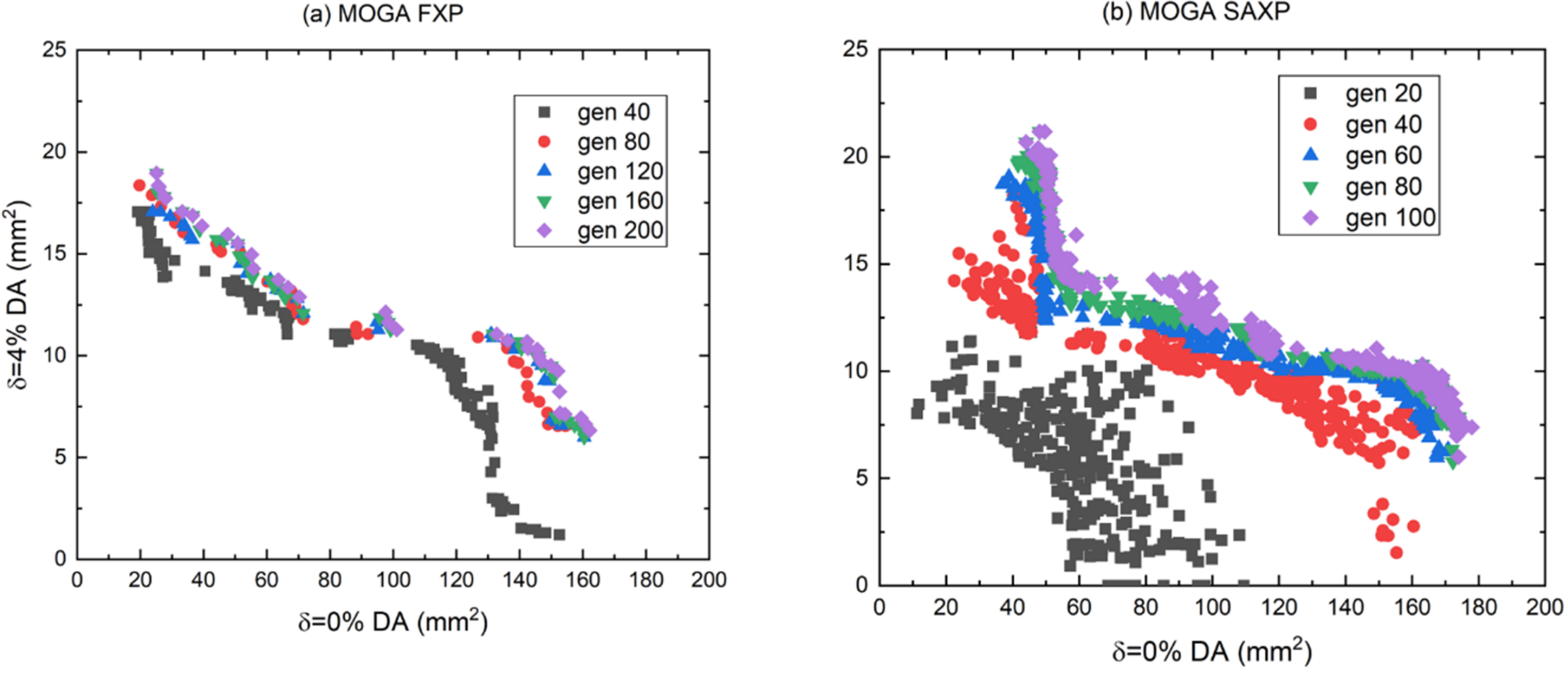
Objectives of all solutions obtained from MOGA using (*a*) FXP at 2*N* generations and (*b*) SXAP with α = 5 at *N* generations where *N* is 20 to 100.
